# The effect of anthocyanins and anthocyanin-rich foods on cognitive function: a meta-analysis of randomized controlled trials

**DOI:** 10.1007/s11357-025-02008-7

**Published:** 2025-12-06

**Authors:** Agnieszka Micek, Justyna Godos, Francesca Giampieri, Maurizio Battino, José L. Quiles, Daniele Del Rio, Pedro Mena, Giuseppe Caruso, Evelyn Frias-Toral, Irma Domínguez Azpíroz, Jianbo Xiao, Nicola Veronese, Mario Siervo, David Vauzour, Zoltan Ungvari, Fabio Galvano, Giuseppe Grosso

**Affiliations:** 1 Statistical Laboratory, Faculty of Health Sciences, Jagiellonian University Medical College, 31-501, Cracow, Poland; 2 Department of Biomedical and Biotechnological Sciences, University of Catania, Catania, Italy; 3 Department of Clinical Sciences, Università Politecnica Delle Marche, 60131 Ancona, Italy; 4 Joint Laboratory On Food Science, Nutrition and Intelligent Processing of Foods, Polytechnic University of Marche, Italy, Universidad Europea del Atlántico, Spain and Jiangsu University, China at Polytechnic University of Marche, 60131 Ancona, Italy; 5 Research Group On Food, Nutritional Biochemistry and Health, Universidad Europea del Atlántico, Isabel Torres 21, 39011 Santander, Spain; 6 International Joint Research Laboratory of Intelligent Agriculture and Agri-Products Processing, Jiangsu University, Zhenjiang, 212013 China; 7 Department of Physiology, Institute of Nutrition and Food Technology “José Mataix Verdú”, Biomedical Research Centre, University of Granada, Avda. del Conocimiento S.N., 18100 Armilla, Spain; 8 Human Nutrition Unit, Department of Food and Drug, University of Parma, 43125 Parma, Italy; 9 Departmental Faculty of Medicine, UniCamillus-Saint Camillus International University of Health and Medical Sciences, Rome, Italy; 10 IRCCS San Camillo Hospital, Venice, Italy; 11 School of Medicine, Universidad Espíritu Santo, Samborondón, 0901952 Ecuador; 12 Division of Research, Texas State University, 601 University Dr, San Marcos, TX 78666 USA; 13 Universidade Internacional Do Cuanza, EN250, Cuito, Angola; 14 Universidad de La Romana, La Romana, 22000 Dominican Republic; 15 Department of Analytical Chemistry and Food Science, Faculty of Food Science and Technology, University of Vigo, Ourense Campus, 32004 Ourense, Spain; 16 Saint Camillus International, University of Health Sciences, Rome, Italy; 17 Curtin-Chulalongkorn Collaborative Centre for Nutrition and Food Research and Education, Curtin University, Perth, WA Australia; 18 Faculty of Health Sciences, School of Population Health, Curtin University, Perth, WA 6102 Australia; 19 Curtin Dementia Centre of Excellence, Enable Institute, Curtin University, Perth, WA 6102 Australia; 20 Curtin Medical Research Institute (CMRI), Curtin University, Perth, WA 6102 Australia; 21 Norwich Medical School, Faculty of Medicine and Health Sciences, University of East Anglia, Norwich, NR4 7TJ UK; 22 Vascular Cognitive Impairment, Neurodegeneration and Healthy Brain Aging Program, Department of Neurosurgery, University of Oklahoma Health Sciences Center, Oklahoma City, OK USA; 23 Stephenson Cancer Center, University of Oklahoma, Oklahoma City, OK USA; 24 Oklahoma Center for Geroscience and Healthy Brain Aging, University of Oklahoma Health Sciences Center, Oklahoma City, OK USA; 25 Department of Health Promotion Sciences, College of Public Health, University of Oklahoma Health Sciences Center, Oklahoma City, OK USA; 26 International Training Program in Geroscience, Doctoral College/Institute of Preventive Medicine and Public Health, Semmelweis University, Budapest, Hungary

**Keywords:** Anthocyanins, Flavonoids, Polyphenols, Cognition, Memory, Brain

## Abstract

The rising prevalence of cognitive disorders highlights the urgent need for effective prevention strategies and therapeutic interventions. While adherence to a balanced diet has been associated with a reduced risk of cognitive decline, emerging evidence underscores the potential role of plant-derived bioactive compounds, such as (poly)phenols, with anthocyanins receiving increasing attention. This meta-analysis aimed to evaluate the effect of anthocyanin-rich interventions on cognitive performance. A systematic search of randomized controlled trials (RCTs) assessing the effects of anthocyanin supplementation and cognitive outcomes identified 59 eligible studies. Overall, anthocyanin intervention significantly improved global cognition (standardized mean difference (SMD) = 0.46, 95% CI = 0.30 to 0.63, *I*^2^ = 0.0%) compared with controls. Domain-specific analyses further revealed significant benefits for visuospatial processing/reasoning and attention (SMD = 0.37, 95% CI = 0.18 to 0.55, *I*^2^ = 76.3%), processing and psychomotor speed (SMD = 0.19, 95% CI = 0.05 to 0.34, *I*^2^ = 64.0%), verbal speed and fluency (SMD = 0.21, 95% CI = 0.03 to 0.39, *I*^2^ = 30.5%), episodic memory (SMD = 0.30, 95% CI = 0.10 to 0.50, *I*^2^ = 75.9%), and working memory (SMD = 0.24, 95% CI = 0.12 to 0.36, *I*^2^ = 46.5%). Collectively, these findings suggest that anthocyanin supplementation may improve multiple cognitive domains. Although these results are promising, further well-designed RCTs are needed to validate these outcomes and consolidate the current evidence base.

**Supplementary Information:**

The online version contains supplementary material available at https://doi.org/10.1007/s11357-025-02008-7.

## Introduction

Cognitive decline and neurodegenerative diseases represent major public health challenges worldwide [1]. The growing aging population and the increasing prevalence of these conditions underline the urgent need for preventive and therapeutic strategies [2]. While genetic predisposition and aging are key determinants in the development of neurodegenerative conditions, accumulating evidence suggests that modifiable lifestyle factors significantly influence brain health [3]. Among these, diet has recently emerged as a critical area of research in relation to cognitive health [4]. Healthy dietary patterns, particularly those rich in bioactive components, have been associated with a reduced risk of cognitive decline and dementia [5]. Plant-derived phytochemicals are of particular interest due to their potential neuroprotective effects and their ability to mitigate the risk of cognitive decline [6]. Traditionally recognized for their antioxidant properties in plants, phytochemicals are increasingly acknowledged for their anti-inflammatory and neuroprotective roles in humans [7, 8]. Regular consumption of fruits, vegetables, whole grains, and other plant-based foods rich in these phytochemicals, has been consistently associated with a reduced risk of cognitive decline and the development of neurodegenerative diseases [9]. Specifically, certain phytochemicals, such as (poly)phenols, have demonstrated the capacity to modulate neuroinflammation, improve vascular health, and enhance neurogenesis, all factors known to contribute to cognitive function and the preservation of brain health [10].

While the neuroprotective effects of various dietary phytochemicals are becoming increasingly recognized, specific classes of compounds, such as anthocyanins, have received substantial attention due to the robust evidence of their positive effects toward vascular and endothelial health [11]. Anthocyanins are a subgroup of flavonoids responsible for the red, blue, and purple coloration in many fruits, vegetables, and flowers. They are abundant in foods such as berries (i.e., blueberries, strawberries, blackberries), grapes, cherries, red cabbage, and eggplant [12]. Anthocyanins have long been valued for their health-promoting properties, supported by strong evidence from observational studies that show their association with reduced risk of cardiovascular-related outcomes [13–15]. However, their potential in supporting cognitive function and reducing the risk of neurodegenerative diseases has been a subject of growing scientific interest. Recently, a summary of evidence from observational studies reported that a higher dietary intake of anthocyanins was inversely associated with cognitive decline, suggesting that the habitual inclusion of anthocyanins in the diet may play a pivotal role against cognitive disorders [16]. The results from preclinical studies support this relation, suggesting that anthocyanins and their metabolites may exert direct neuroprotective effects due to their ability to cross the blood–brain barrier (BBB), reaching the central nervous system and regulating its function through numerous molecular mechanisms, including but not limited to, modulation of adult neurogenesis and synaptic plasticity, inhibiting microglia activation and neuroinflammation as well as regulating mitochondrial function and oxidative stress [17]. Additionally, anthocyanin metabolism may exert indirect neuroprotective effects through modulation of the gut–brain axis [18, 19].

Several studies have previously summarized clinical evidence from randomized controlled trials (RCTs) on the effects of anthocyanins on cognitive outcomes [20–22]. However, such studies have only focused on foods, not taking into account doses of anthocyanins or selected favorable outcomes or specific tests, potentially leading to a certain degree of bias in interpreting the results. Furthermore, since the latest evidence synthesis, additional RCTs have been published. The present study therefore aimed to update and expand the current evidence by systematically and quantitatively evaluating the effects of anthocyanin-rich dietary interventions on general cognition and specific cognitive domains.

## Methods

The conceptualization and execution of this study followed the Preferred Reporting Items for Systematic Reviews and Meta-Analyses (PRISMA) guidelines (Table S1) [23]. The systematic review protocol was registered in the PROSPERO International Prospective Register of Systematic Reviews database (ID: CRD42024557994, at https://www.crd.york.ac.uk/prospero/).

### Search strategy

A systematic search for all RCTs examining the effect of anthocyanin intervention on cognitive outcomes was conducted using PubMed/MEDLINE from its inception to June 2024 and subsequently updated till April 2025. The search strategy was based on a combination of relevant keywords related to anthocyanins, major dietary sources, and cognitive outcomes (Table S2). The reference lists of eligible studies were hand-searched for relevant articles not previously identified. If more than one article reporting results from the same trial was found, only the article including the most comprehensive data was considered eligible for the current meta-analysis. The systematic search and study selection were performed by two independent researchers (A.M. and J.G.).

### Inclusion and exclusion criteria

The eligibility criteria were based on the Population, Intervention, Comparison, Outcomes, and Study (PICOS) design, a framework to formulate eligibility criteria in systematic reviews (Table S3). In particular, studies were eligible if they fulfilled the following inclusion criteria: (i) the design was a randomized controlled trial; (ii) the study evaluated the effect of anthocyanin or anthocyanin-rich intervention based on edible food or extract; (iii) the study reported data on cognitive function, in particular general cognition and/or specific cognitive domains. On this last matter, most outcomes assessed in the studies are somehow interconnected and represent the whole (or at least a significant part) of an individual’s cognitive functioning. Based on a reasonable approach, the outcomes were categorized as follows: (i) “Memory”, further distinguishing “Working” and “Episodic” memory, including those tests specifically assessing the functionalities of an active mnemonic task relevant for a short window of time (including word, picture, and numeric recognition and memory) and longer-lasting memory related to experience (including verbal recalls), respectively [24]; (ii) “Visuospatial processing/reasoning and attention” tests were grouped as they share similar characteristics and recruit overlapping brain regions emphasizing the static (non-motor) processes encoding, maintenance, and retrieval [25]; (iii) “Processing and psychomotor speed” tests were grouped to emphasize the “motor” aspect of processing and the “time” component of the process (from receiving the task to executing), as both processing the intention to react and the following motor action are hypothesized to be strictly related to the same brain structural regions [26]; and (iv) “Verbal speed, fluency, and accuracy” outcomes included those tests related to executive functions specifically concerning processing, retrieving, and producing verbal information [27]. Exclusion criteria were the following: (i) studies assessing immediate effects of the intervention; (ii) studies involving individual or limited administrations; and (iii) studies on children and adolescents, pregnant women, or patients with end-stage degenerative diseases. Studies that provided insufficient statistical data were also not included.

### Data extraction and study quality assessment

Data from all the eligible studies was extracted using a standardized electronic form. The following information was collected: first author name and publication year, study design and location, population characteristics, number of participants, population age and sex, intervention duration, type of intervention and its main characteristics, type of control, details on the outcome of interest, and measures needed to calculate size effects for each intervention at the beginning and at the end of the trial.

The quality of each included study was assessed by two authors (A.M. and J.G.) using the revised Cochrane risk-of-bias tool for randomized trials (RoB 2) [28]. Briefly, the tool consists of five domains evaluating: (i) bias arising from the randomization process, (ii) bias due to deviations from intended interventions, (iii) bias due to missing outcome data, (iv) bias in outcome measurement, and (v) bias in selection of the reported results, and additionally for cross-over trials the tool evaluates bias arising from period and carryover effects [28]. Based on the individual domain risk-of-bias judgments, the overall risk-of-bias judgment is defined as “low risk of bias,” “some concerns,” or “high risk of bias” [28].

### Statistical analysis

A meta-analysis was performed to quantify the pooled effect of anthocyanins and anthocyanin-rich foods on cognitive outcomes. Analyses employed the so-called raw score metric to calculate standardized mean differences (SMDs). Next, effect sizes were harmonized within the same outcome, taking into account variability within each intervention group. As recommended by Tsai et al. 2021 [29], in studies that presented estimates for several tests (more than one) in a specific cognitive domain, the most representative test was selected through discussion and consensus. Additionally, to assess the robustness of the findings, sensitivity analyses were conducted using alternative selections of psychological tests within each cognitive domain in each study, considering both the most favorable and the most unfavorable effect size scenarios. As a complementary approach, studies reporting multiple outcomes within the same domain were aggregated into a single effect size, with SMDs averaged and their variances adjusted to account for correlations between outcomes (conservatively imputed at a level of 0.5 based on conventional use in scientific literature). A random-effects model with the DerSimonian and Laird estimator of between-study variability was implemented. Statistical heterogeneity was assessed by the *I*^*2*^ statistic and was formally complemented by the Cochran Q-test. Pooled results were reported as SMDs with 95% confidence intervals (CIs) and 2-sided *P* values. In reports that failed to provide sufficient data for computing effect size estimates properly, accounting for the paired nature of the design, the correlation between measurements before and after each intervention was conservatively imputed at a level of 0.5. Sensitivity analyses further explored assumed correlations of *r* = 0.2 and 0.8. The effect of omitting one study at a time on the pooled estimates was checked in the influential analysis. The small-study effect and possible publication bias were assessed by verifying asymmetry in funnel plots and using Egger’s regression test. A trim-and-fill method was then applied to adjust for the number of missing studies. A P-value less than 0.05 was considered statistically significant. All analyses were performed with R version 4.4.1 (Development Core Team).

## Results

The process of systematic search and study selection is presented in Fig. S1. The systematic search identified 625 potential articles for screening; after title and/or abstract evaluation, 527 articles were excluded, leaving 98 articles for full-text evaluation. Finally, after excluding 39 articles that did not meet the eligibility criteria, a total of 59 articles reporting results from randomized controlled trials on anthocyanin or anthocyanin-rich interventions and cognitive outcomes were included in meta-analyses.

### Main characteristics of the included studies

The main characteristics of the studies included are presented in Table 1 [30–56, 58–88, 88]. A total of 9 studies were cross-over trials, and 50 had a parallel design. Twenty-two trials were conducted in European countries, 24 in North and South America, with 22 specifically involving US cohorts, 12 included populations from Asia and Oceania, and one study was multicentric. Most studies included older adults: specifically, the mean age of participants exceeded 65 years in 32 studies, ranged from 55 to 65 years in 14 studies, ranged from 45 to 55 years in 4 studies, and it was below 45 years in 8 studies (1 study did not report information on the mean age of participants). The majority of the studies (*n* = 53) involved both sexes, while 3 studies only included females, and 3 studies only included males. The duration of intervention differed between studies and lasted from 1 to 2 weeks (*n* = 6 studies), through 1 to less than 3 months (*n* = 16 studies), 3 to 4 months (*n* = 23 studies), 6 months (*n* = 13 studies), and 12 months (*n* = 1 study). Concerning the health status of participants, 34 trials investigated healthy individuals, 22 trials included individuals with mental or cognitive problems, and 3 trials investigated cardiac, prostate cancer, and ischemic stroke patients.

**Table 1:** The main characteristics of the studies included in the meta-analysis

Author, year, country	Age range	Design	Participants characteristics	N, sex, mean age	Foll. Up&washout	Intervention	Daily anthocyanin supplementation	Comparison	Cognitive domains
Crews, 2005, USA	60 +	DB, P–C, PA	Healthy, volunteer men and women aged ≥ 60 years	47 (nC: 23, nI: 24) MF, 69.3 y	6 w	Low-calorie cranberry juice product, containing 27% juice/volume and sweetened with sucralose (16 oz/dose) taken orally twice daily (i.e., 32 oz/day)	NR	Placebo matched in appearance, smell, taste, and vitamin C content)	ProcPsychSpeed, WorkMem
Krikorian, 2010, USA	NA	DB, P–C, PA	Non-demented older adults with memory decline (aged at least 65 years)	12 (nC: 32, nI: 31) MF, 78.2 y	12 w	Concord grape juice (444–621 ml/d: 6–9 ml/kg/d) (1 × 3)	NR	Beverage formulated to look and taste like grape juice but containing no juice or natural polyphenols	EpisMem, VisspatProcAtten
Krikorian, 2012, USA	68 +	DB, P–C, PA	Older people (aged at least 68 years) with mild cognitive impairment	21 (nC: 11, nI: 10) MF, 76.5 y	16 w	Concord grape juice (355–621 ml/d: 6.3–7.8 ml/kg/d) (1 × 3)	151–264 mg of anthocyanin	The placebo beverage contained no juice or polyphenolic compounds	EpisMem
Bookheimer, 2013, USA	NA	DB, P–C, PA	Non-demented volunteers with mild memory complaints	28 (nC: 13, nI: 15) MF, 62.6 y	28 d	Pomegranate juice (237 ml × 1/d)	NR	Placebo drink imitating the taste and appearance of pomegranate juice	WorkMem
Ropacki, 2013, USA	NA	DB, P–C, PA	Patients undergoing elective coronary artery bypass graft and/or valve surgery	10 MF, 66.3 y	7 w	Pomegranate extract capsules (one in the morning and one in the evening, each 1 g)	186 mg of anthocyanin	Placebo capsules (two) that looked identical to the pomegranate pills but contained no pomegranate ingredients	WorkMem
Small, 2014, USA	65 +	DB, P–C, PA	Non-demented elderly aged 65–85 years	105 (nC: 53, nI: 52) MF, 73.6 y	2 m	Pill-based nutraceutical (NT-020) containing a formulation of blueberry, carnosine, green tea extract (95% polyphenols), 2000 IU vitamin D3, VitaBlue (40% polyphenolics, 12.5% anthocyanins from blueberries), 40 mg Biovin, grape polyphenolics, including 5% resveratrol (1 × 2)	NR	Placebo matched pills	ProcPsychSpeed, VerbMemFluen, WorkMem
Schrager, 2015, USA	60 +	NR, P–C, PA	Older healthy individuals (aged at least 60 years)	23 (nC: 7, nI: 13) MF, 69.1 y	6 w	Frozen highbush blueberries (Vaccinium corymbosum) (2 cups (0.47 kg)/d, fairly even distribution ingestion over the course of each day)	NR	Carrot juice drink (not containing anthocyanins)	ProcPsychSpeed
Lamport, 2016, UK	40–50	DB, P–C, CO	Healthy, middle-aged working mothers of preteen children (aged 40–50 y)	25 F, 43.0 y	2 × 12 w (4 w)	Concord grape juice (335 ml/d details of distribution of ingestion not reported)	167 mg of anthocyanin	Placebo drink (matched for energy, appearance, taste, volume, carbohydrate content, all sugars)	ProcPsychSpeed, VisspatProcAtten, WorkMem
Bowtell, 2017, UK	65 +	DB, P–C, PA	Healthy individuals (aged at least 65 years)	26 (nC: 14, nI: 12) MF, 68.3 y	12 w	Blueberry concentrate (30 ml × 1/d)	387 mg of anthocyanin	Isoenergetic placebo	ProcPsychSpeed, VerbMemFluen, VisspatProcAtten, WorkMem
Calapai, 2017, Italy	55–75	DB, P–C, PA	Healthy older adults aged 55–75 years	111 (nC: 54, nI: 57) MF, 66.9 y	12 w	V. vinifera-based dietary supplement capsule Cognigrape V. vinifera fruit extract containing also 30–40% maltodextrin (250 mg/day) (1 × 1)	4–5% w/w of anthocyanin	Placebo supplement capsule (composed only of maltodextrin)	GenCogn, VerbMemFluen, VisspatProcAtten, WorkMem
Kent, 2017, Australia	70 +	DB, P–C, PA	Older adults with mild to moderate dementia (aged at least 70 years)	49 (nC: 25, nI: 24) MF, 79.8 y	12 w	Cherry juice (200 ml × 1/d)	138 mg of anthocyanin	Apple juice (200 ml/d)	ProcPsychSpeed, VerbMemFluen, WorkMem
Lee, 2017, US	65 +	DB, P–C, PA	Individuals with mild cognitive impairment (aged at least 65 years)	10 (nC: 5, nI: 5) MF, 72.2 y	6 m	Grape formulation (freeze-dried grape powder reconstituted in 237 ml of water) (36 g × 2/d)	330.4 mg of anthocyanin	Placebo formulation free of polyphenols (matched in appearance, flavor, smell, volume and content of fructose and glucose)	EpisMem, GenCogn, ProcPsychSpeed, VerbMemFluen, VisspatProcAtten, WorkMem
Nilsson, 2017, Sweden	50–70	NB, C, CO	Healthy older (aged between 50–70 years) non-smoker volunteers with a normal to slightly increased BMI	40 (nC: 20, nI: 20) MF, 63.0 y	2 × 5 w (5 w)	Berry beverage consisted of a mixture of Swedish berries (150 g blueberry, 50 g elderberry, 50 g lingonberry, 50 g strawberry, 50 g blackcurrant, and 6 g tomato powder) (200 ml × 3/d)	249 mg of anthocyanin	Beverage not containing anthocyanins (matched for low-molecular weight carbohydrates and pH)	ProcPsychSpeed, VisspatProcAtten, WorkMem
Boespflug, 2018, USA	68 +	DB, P–C, PA	Older adults with age-related memory decline (aged at least 68 years)	16 (nC: 8, nI: 8) MF, 78.0 y	16 w	Powder prepared from whole freeze-dried blueberry fruit (Vaccinium) (12.5 g × 2/d)	269 mg of anthocyanin	Placebo powder matched for color, taste, and sugar content	WorkMem
McNamara, 2018, USA	62 +	DB, P–C, PA	Individuals with subjective cognitive decline (aged 62 to 80 years old)	39 (nC: 20, nI: 19) MF, 68.0 y	24 w	Freeze-dried blueberry powder (Vaccinium sp.) + placebo oil (12.5 g × 2/d)	269 mg of anthocyanin	Placebo powder + placebo oil	EpisMem, ProcPsychSpeed, VerbMemFluen
Miller, 2018, USA	60–75	DB, P–C, PA	Subjects 60–75 years old with age-related motor and cognitive decline	37 (nC: 19, nI: 18) MF, 67.6 y	90 d	Freeze-dried blueberry (lyophilized Tifblue blueberry) (12 g × 2/d)	460.8 mg of anthocyanin	Placebo powder, isocaloric, blueberry flavored, mainly consisting of maltodextrin and fructose	EpisMem
Traupe, 2018, Italy	50–75	SB, C, PA	Middle-aged (50–75 years) adults undergoing general anesthesia (during prostatectomy due to cancer)	26 (nC: 13, nI: 13) M, 66.5 y	2 w	Blueberry juice and pulp (Vaccinium myrtillus) (170 ml × 3/d)	NR	Control group—no placebo drink	ProcPsychSpeed, VisspatProcAtten, WorkMem
Whyte, 2018, UK	65–80	DB, P–C, PA	Older healthy volunteers (aged 65–80 years) with subjective self-reported memory complaints	112 (nC: 27, nI: 28/29/28) MF, 70.8 y	6 m	Wild blueberry (WBB) formulation 2000 mg/d (encapsulated) (2 × 1) containing 900 mg/d WBB powder (P500) or 1800 mg/d WBB powder (P1000) or 200 mg/d WBB extract (E111)	2.7/5.4/14 mg of anthocyanin	Placebo consisting of maltodextrin and food dye	EpisMem
Bellone, 2019, USA	18–89	DB, P–C, PA	Ischemic stroke inpatients receiving comprehensive rehabilitative care aged 18–89 years old	14 MF, 59.0 y	1 w	Pomegranate capsules each containing 1 g of a concentrated blend of polyphenols (twice per day in the morning and night)	NA	Placebo pills similar to the pomegranate pills except that they contained only lactose	GenCogn, ProcPsychSpeed, VerbMemFluen, VisspatProcAtten, WorkMem
Bensalem, 2019, Canada and France	60–70	DB, P–C, PA	Older, non-obese healthy individuals (60–70 years-old)	190 (nC: 98, nI: 92) MF, 64.7 y	24 w	Polyphenol-rich extract from grape (Vitis vinifera L.) and wild blueberry (Vaccinium angustifolium Aiton.), 600 mg/d, encapsulated (1 × 2)	0.78 mg of anthocyanin	Placebo consisting of maltodextrin	EpisMem, WorkMem
Chai, 2019, USA	65–80	NB, C, PA	Older subjects with normal cognitive function between the ages of 65–80	34 (nC: 17, nI: 17) MF, 64.7 y	12 w	Tart cherry juice, 480 ml/d (68 ml Montmorency tart cherry juice concentrate diluted with 412 ml water)	NR	Control drink (unsweetened black cherry flavored Kool-Aid (Kraft Foods, United States) mixed with water)	EpisMem, ProcPsychSpeed, VisspatProcAtten, WorkMem
Joo, 2019, South Korea	50 +	DB, P–C, PA	Participants aged 50 years or older with subjective memory impairment but with no clear impairment on objective psychometric testing	48 (nC: 25, nI: 23) MF, 63.9 y	12 w	500 mg black rice (cyanidin-3-glucoside-rich Oryza sativa L.) extract capsule (50% Oryza sativa L. extract powder, 40% crystalline cellulose 9% glucose, and 1% malic acid): 2 capsules three times a day	19.08 mg of C3G	Placebo extract capsules (500 mg) identical in size and appearance (100% crystalline cellulose)	EpisMem, GenCogn, ProcPsychSpeed, VerbMemFluen, VisspatProcAtten
Ahles, 2020, Netherlands	40–60	DB, P–C, PA	Healthy, middle-aged, overweight adults aged 40–60 years	101 (nC: 32, nI: 34/35) MF, 53.0 y	24 w	90 mg Aronia melanocarpa (AM90) or 150 mg Aronia melanocarpa (AM150) (1 × 1 capsule ingested with 200 mL water)	16/27 mg of anthocyanin	Maltodextrin capsule (150 mg)	ProcPsychSpeed, VisspatProcAtten
Cook, 2020, UK	NA	DB, P–C, CO	Community dwelling, physically active older adults, free from any injuries	14 MF, 69.0 y	2 × 7 d (7 d)	Concentrated New Zealand blackcurrant (NZBC) extract (600 mg/d) (2 capsules daily)	105 mg of anthocyanin	Placebo (microcrystalline cellulose)	EpisMem, ProcPsychSpeed, VisspatProcAtten, WorkMem
Gibson, 2020, New Zealand	NA	DB, P–C, CO	Rugby league players	23 MF, 28.0 y	2 × 1 w (10 d)	Blackcurrant-based nootropic-drink containing blackcurrant juice, apple juice, water, flavors, blackcurrant extract, decaffeinated green tea extract, citric, l-theanine, pine bark extract (supplement Arepa®) 300 mL	465 mg of anthocyanin	An iso-caloric, appearance and taste matched inactive control beverage (placebo)	ProcPsychSpeed
Helmer, 2020, USA	42–65	DB, P–C, PA	U.S. veterans of Gulf War aged 42–65 years diagnosed with Gulf War Illness	36 (nC: 18, nI: 18) MF, 53.0 y	24 w	Concord Grape Juice (CGJ, 4 oz in weeks 0–2, 8 oz in weeks 3–4, 16 oz in weeks 5–24, about 424 ml on average)	173 mg of anthocyanin	The placebo beverage containing no juice or polyphenolic compounds and composed of fructose, corn syrup, natural grape essence and matched by color, taste, calories and sugar with CGJ	EpisMem, GenCogn, ProcPsychSpeed, VisspatProcAtten
Igwe, 2020, Australia	55 +	SB, C, CO	Older adults (aged 55 years or older) without cognitive impairment	31 MF, 70.0 y	2 × 8 w (4 w)	Queen Garnet plum (QGP) nectar (200 mL)	10 mg of anthocyanin	Control raspberry cordial beverage (with 0.8 mg anthocyanidins)	ProcPsychSpeed, VerbMemFluen, WorkMem
Krikorian, 2020, USA	68 +	DB, P–C, PA	Older adults (aged 68 years or older) with mild cognitive impairment	37 (nC: 21, nI: 16) MF, 77.1 y	16 w	Freeze-dried blueberry fruit powder (24 g/d: 2 × 12 g of powder in the morning and in the evening)	258 mg of C3G	Placebo powder including artificial purple and red coloring, artificial blueberry flavor, natural blueberry flavor, maltodextrin, fructose, glucose, and citric acid	EpisMem, ProcPsychSpeed, VerbMemFluen, VisspatProcAtten
Mirheidary, 2020, Iran	18–30	NB, C, PA	Healthy students aged 18 to 30 years old	53 (nC: 17, nI: 36) MF, 20.8 y	4 w	A specific type of dried grapes “maviz”, 25 g (delivered in packs) orally in the morning	NA	No intervention (group without "maviz" supplementation)	WorkMem
Siddarth, 2020, USA	50–75	DB, P–C, PA	Normal aging or mild cognitive impairment, nondemented subjects (aged 50–75 y)	200 (nC: 102, nI: 98) MF, 60.4 y	12 m	Pomegranate juice (236.5 ml/d)	93 mg of anthocyanin	Placebo drink not containing polyphenols and matched for flavor, color, sugar, and acidity level	VisspatProcAtten, WorkMem
Bohn, 2021, Norway	67 +	DB, P–C, PA	Men of Norwegian ethnicity between 67 and 77 years of age with subjective memory impairment	60 (nC: 30, nI: 30) M, 71.5 y	9 w	50/50 mix of bilberry (Vaccinium Myrtillus) and red grape (Vitis Vinifera) juice twice a day 330 ml	NR	Iso-caloric placebo juice (6.25 g sucrose, 6.25 g maltodextrine, 1.3 g citric acid, 2.5 g Carmine solution E120, 0.025 g blueberry aroma, Potassium sorbate E202 and water)	EpisMem, ProcPsychSpeed, VisspatProcAtten
De Oliveira, 2021, Brazil	NA	NB, C, PA	People diagnosed with idiopathic Parkinson's Disease and physically inactive for at least one month	19 (nC: 9, nI: 10) MF, 67.0 y	30 d	Aquatic exercise program (ACQ-EXE, twice a week, lasting one hour each session) plus grape juice (GJ from Vitis labrusca) consumption (2 packages daily, each 200 ml)	NR	Aquatic exercise program (ACQ-EXE, twice a week, lasting one hour each session) without grape juice	GenCogn
Miller, 2021, USA	60–75	DB, P–C, PA	Older men and women (60–75 years)	37 (nC: 19, nI: 18) MF, 67.6 y	90 d	Freeze-dried strawberries (lyophilised, standardised blend sourced from equal parts of Albion, San Andreas, Camino Real and Well-Pict 269 varieties, twice daily 12 g)	NR	Colour-matched, isoenergetic, placebo powder comprised of maltodextrin, fructose, dextrose, sucrose, cellulose and xanthan gum, food starch, citric and malic acids, sugar beet fibre, dipotassium phosphate, potassium citrate, silicon dioxide, natural strawberry flavour and food colouring (12 g twice daily)	EpisMem, VisspatProcAtten
Rosli, 2021, Malaysia	45–59	DB, P–C, PA	Middle-aged women (aged 45–59 years) with signs of poor cognitive function	31 (nC: 15, nI: 16) F, 78.2 y	30 d	Polyphenols-rich tropical fruit juice (TP 3-in-1™) formulation consisted of a mixture of tropical fruit: pomegranate concentrate with guava and roselle extract, 1500 ml/d (3 × 500 ml/d)	194.1 mg/d of anthocyanin	Placebo beverage	ProcPsychSpeed, WorkMem
Flanagan, 2022, UK	50–80	DB, P–C, PA	Healthy male and female older adults aged between 50 and 80 years	60 (nC: 31, nI: 29) MF, 65.6 y	12 w	Freeze dried cranberry powder (two sachets per day (each 4.5 g), one in the morning and one in the evening incorporated in food or beverages)	59 mg of anthocyanin	Placebo powder matched the active cranberry powder for taste, colour, total sugar, fructose, calories and contained a blend of water, maltodextrin, citric acid, artificial cranberry flavour, fructose, red colour (Lorann oils) and grape shade that had been freeze-dried	ProcPsychSpeed, VerbMemFluen, VisspatProcAtten, WorkMem
Kimble, 2022, UK	NA	DB, P–C, PA	Non-smoking adults	50 (nC: 25, nI: 25) MF, 48.0 y	12 w	Tart Montmorency cherries concentrate diluted in water (30 ml × 2/d) (each 30 ml serving diluted in 240 ml of water)	22.2 mg of anthocyanin	An isoenergetic placebo	VisspatProcAtten
Krikorian, 2022, USA	50–65	DB, P–C, PA	Middle-aged (aged 50 to 65 years) sample of non-diabetic, insulin-resistant, overweight participants with subjective cognitive decline and elevated risk for future dementia	27 (nC: 14, nI: 13) MF, 56.4 y	12 w	Blueberry powder (a daily dosage of 0.5 c whole-fruit equivalent administered once each day)	NR	Placebo powder	EpisMem, VerbMemFluen
Nakamura, 2022, Japan	50–75	NR, P–C, PA	Healthy Japanese adults aged from 50 to 75 years	24 (nC: 12, nI: 12) MF, 59.1 y	12 w	WFBSK: mixture of unpolished super-hard rice (SHBR), wax-free unpolished black rice (WFBBR), ordinary non-polished rice (KBR) (blending ratio 4:4:2), adding 2.5% waxy black rice bran (WBB) and 0.3% rice oil (one package daily 190.0 g)	15.2 mg of anthocyanin	White rice (200 g daily, one package)	ProcPsychSpeed, VerbMemFluen, VisspatProcAtten
Aarsland, 2023, Norway	60–80	DB, P–C, PA	Older participants (aged 60–80 years) with MCI or cardiometabolic disorders known to be associated with increased risk of cognitive decline and dementia	206 (nC: 100, nI: 106) MF, 69.3 y	24 w	Medox capsules (each capsule contains 50% Maltodextrin Glucidex IT 19, 50% bilberry (V. myrtillus) and blackcurrant (R. nigrum) extract powder with 80-mg anthocyanin citrates) (1 × 2)	160 mg of anthocyanin	Placebo capsules (91% maltodextrin and 9% citric acid)	EpisMem, ProcPsychSpeed, VisspatProcAtten, WorkMem
Cheatham, 2023, USA	65–80	DB, P–C, PA	Adult volunteers, 65–80 years, with age-related mild cognitive decline	65 (nC: 36, nI: 29) MF, 72.3 y	24 w	A lyophilized wild blueberry powder (35 g per day)	411.25 mg of anthocyanin	A calorie-matched placebo powder (35 g per day)	VisspatProcAtten
Krikorian, 2023, USA	50–65	DB, P–C, PA	Overweight, middle-aged subjects with complaints of mild cognitive decline, 50 to 65 years old	30 (nC: 15, nI: 15) MF, 56.5 y	12 w	Strawberry powder (from freeze-dried, and milled fruit) (1 × 13 g/d derived from 130 g whole fruit) mixed with water	36.8 mg of anthocyanin	Placebo powder	EpisMem
Lopresti, 2023, France	60 +	DB, P–C, PA	Volunteers aged 60 to 80 years with mild cognitive impairment (experiencing self-reported difficulties with attention and memory, and scoring between 13 and 18 on the MoCA-BV)	120 (nC: 56, nI: 64) MF, 67.9 y	6 m	Polyphenol-rich blend of grape and wild blueberry extracts in capsules (150 mg of Memophenol™, twice daily)	NR	Placebo capsules (containing maltodextrin) identical in appearance, matched for color, shape, size, smell, and taste	EpisMem, ProcPsychSpeed, VisspatProcAtten, WorkMem
Wattanathorn, 2023, Thailand	45–65	DB, P–C, PA	Middle-aged participants with normal weight and normal cognitive ability aged between 45 and 65 years old	69 (nC: 23, nI: 23/23) MF, 50.8 y	8 w	Functional soup containing “Anthaplex” (mixture of germinated purple corn and rice berry) either at 2 (D2) or 4 g (D4) per serving per day, mixed with JWF 1/2564 ingredient (trade secret) (1 × 120 ml/d in the morning)	13.8/16.6 mg of anthocyanin	Placebo mixture matched for appearance, taste, volume, and calories (containing purple corn)	EpisMem, ProcPsychSpeed, VisspatProcAtten, WorkMem
Wood, 2023, UK	65–80	DB, P–C, PA	Healthy older individuals aged 65–80 years	61 (nC: 27, nI: 27) MF, 70.1 y	12 w	Freeze-dried wild blueberry (WBB) powder (26 g/d)	302 mg of anthocyanin	Matched placebo powder (26 g/d)	EpisMem, ProcPsychSpeed, WorkMem
Ahles, 2024, Netherlands	18–35	DB, P–C, CO	Non-obese healthy young adults (aged 18–35 years)	35 MF, 25.0 y	2 × 1 w (2 w)	Aronia melanocarpa extract (AME) (750 mg/d, 3 × 1 capsules diluted with 200 ml water)	180 mg of anthocyanin	Placebo capsules (containing cellulose)	EpisMem, ProcPsychSpeed
Amone, 2024, Italy	55 +	DB, P–C, PA	Healthy males and females aged more than 55 years old with normal cognitive function	96 (nC: 48, nI: 48) MF, 60.4 y	84 d	Extract of Vitis vinifera (L.) (250 mg) supported on maltodextrins [30–40%], pregelatinized corn starch (87.75 mg), vegetable magnesium stearate (1.35 mg), talc (0.45 mg), and colloidal silica (0.45 mg) (once daily one capsule)	4–5% of anthocyanin	Capsule of the same appearance of the active product and containing maltodextrin (250 mg), pregelatinized corn starch (87.75 mg), vegetable magnesium stearate (1.35 mg), talc (0.45 mg), and colloidal silica (0.45 mg)	EpisMem, GenCogn, ProcPsychSpeed, VerbMemFluen, VisspatProcAtten, WorkMem
Borda, 2024, Norway	60–80	DB, P–C, PA	Individuals aged 60–80 years with Mild Cognitive Impairment (MCI) and with Cardiometabolic disorders (CMD)	201 (nC: 98, nI: 103) MF, 68.9 y	24 w	Capsules including a standardized nutraceutical product containing 80 mg of naturally purified anthocyanins from bilberry (Vaccinium myrtillus) and black currant (Ribes nigrum) (two capsules twice daily)	320 mg of anthocyanin	The placebo capsules, indistinguishable in appearance, contained 91% maltodextrin and 9% citric acid	EpisMem
Curtis, 2024, UK	50–75	DB, P–C, PA	Elderly adults with overweight and obesity and MetSm2), aged 50–75 y	138 (nC: 39, nI: 39/37) MF, 62.9 y	6 m	Freeze-dried blueberry powder (26 g of freeze-dried blueberry sachet, or a hybrid treatment sachet that combined 13 g freeze-dried blueberries and 13 g placebo material)	182/364 mg of anthocyanin	Placebo in powdered, freezedried form isocaloric and matched for carbohydrate content	EpisMem, ProcPsychSpeed, VisspatProcAtten, WorkMem
Curtis, 2024, Columbia	50 +	DB, P–C, PA	Adults with MCI aged 50 + years	24 (nC: 13, nI: 11) MF, 76.3 y	6 m	American elderberry juice (5 mL orally 3 times a day)	47.7 mg of anthocyanin	Placebo–control juice contained flavored liquid with no nutritional content	EpisMem, GenCogn, VerbMemFluen, VisspatProcAtten
Gillies, 2024, New Zealand	18–45	DB, P–C, CO	Healthy, non-obese young and middle-aged females (18–45 y)	38 F, 29.8 y	2 × 4 w (2 w)	Flavonoid-rich blackcurrant juice and extracts (300 ml containing 150 mg Enzogenol, and 200 mg L-theanine)	151 mg of anthocyanin	Control beverage matched for taste, appearance, macronutrient and vitamin C	GenCogn, ProcPsychSpeed, VisspatProcAtten, WorkMem
Lazou-Ahrén, 2024, Sweden	70 +	DB, P–C, PA	The healthy participants of both genders, aged at least 70 years with low grade systemic inflammation	44 (nC: 22, nI: 22) MF, 73.2 y	4 w	A powder with 10 g per sachet of probiotic strain Lactiplantibacillus plantarum HEAL9 with the addition of a mixture of freeze-dried blackberries and black currants (3 g of freeze-dried powder each berry), once daily after mixing with sour milk or pouring over the breakfast flakes	NR	Placebo powder with red beet extract and flavor added to give the same appearance, taste, and texture and with maltodextrin as a filler	ProcPsychSpeed
Lopresti, 2024, Australia	40–75	DB, P–C, PA	Healthy males and females aged between 40 and 75 years, subjectively reporting memory problems, non-smokers, with a body mass index (BMI) between 18 and 35 kg/m2, no plans to start new treatments	100 (nC: 50, nI: 50) MF, 59.4 y	12 w	Nutraceutical as 2 softgel capsules once daily with food (9 mg astaxanthin, 250 mg grape juice extract, and 12 mg vitamin E daily)	NR	Placebo softgel capsules identical in appearance, and matched for size, shape, color, and excipients	EpisMem, ProcPsychSpeed, VisspatProcAtten, WorkMem
Güçer Öz, 2024, Turkey	65 +	NB, C, PA	Individuals over the age of 65 who were diagnosed with mild-to-moderate AD	39 (nC: 19, nI: 20) MF, 80.5 y	12 w	Morus nigra concentrate (20 g per day)	21.8 mg of anthocyanin	No intervention	GenCogn
Velichkov, 2024, UK	18–24	DB, P–C, PA	Emerging adults with self-reported symptoms of depression, 18–24 years of age	60 (nC: 30, nI: 30) MF, 20.0 y	6 w	Drink prepared by mixing 250 ml water with 22 g freezedried wild lowbush blueberries (Vaccinium angustifolium), every morning 1 drink	121 mg of anthocyanin	Blueberry-flavoured placebo drink matched for carbohydrates and fibre	ProcPsychSpeed
Arbizu, 2025, USA	18 +	SB, P–C, PA	Adults aged 18 years and older with BMI between 30 and 40 and no history of chronic diseases or intestinal disorders	40 (nC: 21, nI: 19) MF, NR	30 d	Dark sweet cherry concentrated juice (50 mL) supplemented with 3 g of dark sweet cherry powder and reconstituted with water up to 200 mL twice daily	70.21 mg of C3G	Placebo concentrated drink	ProcPsychSpeed, VisspatProcAtten, WorkMem
Carrillo, 2025, Spain	18–65	DB, P–C, CO	University students and staff aged between 18 and 65 years	92 MF, 34.0 y	2 × 16 w (4 w)	Encapsulated concentrate of fruit, vegetable, and berry juice powders (3 capsules daily with breakfast and 3 with dinner) (apple, carrot, grape, pomegranate, orange, pineapple, blueberry, lingonberry, American bilberry, blackberry, cabbage, garlic, myrtle, mango, raspberry, acerola, peach, date, parsley, broccoli, spinach, kale, tomato, elderberry, blackcurrant, plum, and beet)	NR	Placebo formulated from microcrystalline cellulose and matched the active product in both appearance and dosage	GenCogn, ProcPsychSpeed
Farhat, 2025, UK	55–70	NB, C, PA	Older adults aged 55–70 years	78 (nC: 37, nI: 41) MF, 61.3 y	12 w	Pomegranate extract (PE) capsules supplementation (740 mg) (75% punicalagins and 1.3% ellagic acid)	NA	Placebo capsules (maltodextrin) identical in appearance	ProcPsychSpeed, VisspatProcAtten, WorkMem
Musich, 2025, USA	50 +	DB, P–C, PA	Patients with mild cognitive impairment aged 50 + years	24 (nC: 13, nI: 11) MF, 76.3 y	6 m	Elderberry juice (5 mL three times a day)	47.7 mg of C3G	Placebo-control juice	GenCogn, VisspatProcAtten
Naderi, 2025, Iran	< 25	DB, P–C, CO	Iranian male national level rowers	9 M, 19.0 y	3 × 7 d (14 d)	New Zealand blackcurrant extract (600 mg CurraNZ daily including 210 mg anthocyanin) encapsulating in 500 mg Black capsules (two capsules daily during breakfast)	210 mg of anthocyanin	Two placebo black capsules matched in the colour	ProcPsychSpeed

Abbreviations: *VisspatProcAtten* Visuospatial processing/reasoning and attention, *C* controlled, *C3G*cyanidin 3-glucoside, *CO*cross-over, *d*days, *DB*double blind, *EpisMem*Episodic memory, Ffemales, *GenCogn*General cognition, *M*males, *m*months, *MF*males and females, *nC*number of participants in control group, *nI*number of participants in intervention group, *No*number of participants, *NR*not reported, *PA*parallel, *P-C*placebo controlled, *ProcPsychSpeed*Processing and psychomotor speed, *SB*single blind, *TB*triple blind, *VerbMemFluen*Verbal speed and fluency, *w*weeks, *WorkMem*Working memory, *y*years

^&^For cross-over trials, the duration of the washout period is given in parentheses

**Fig. 1:**
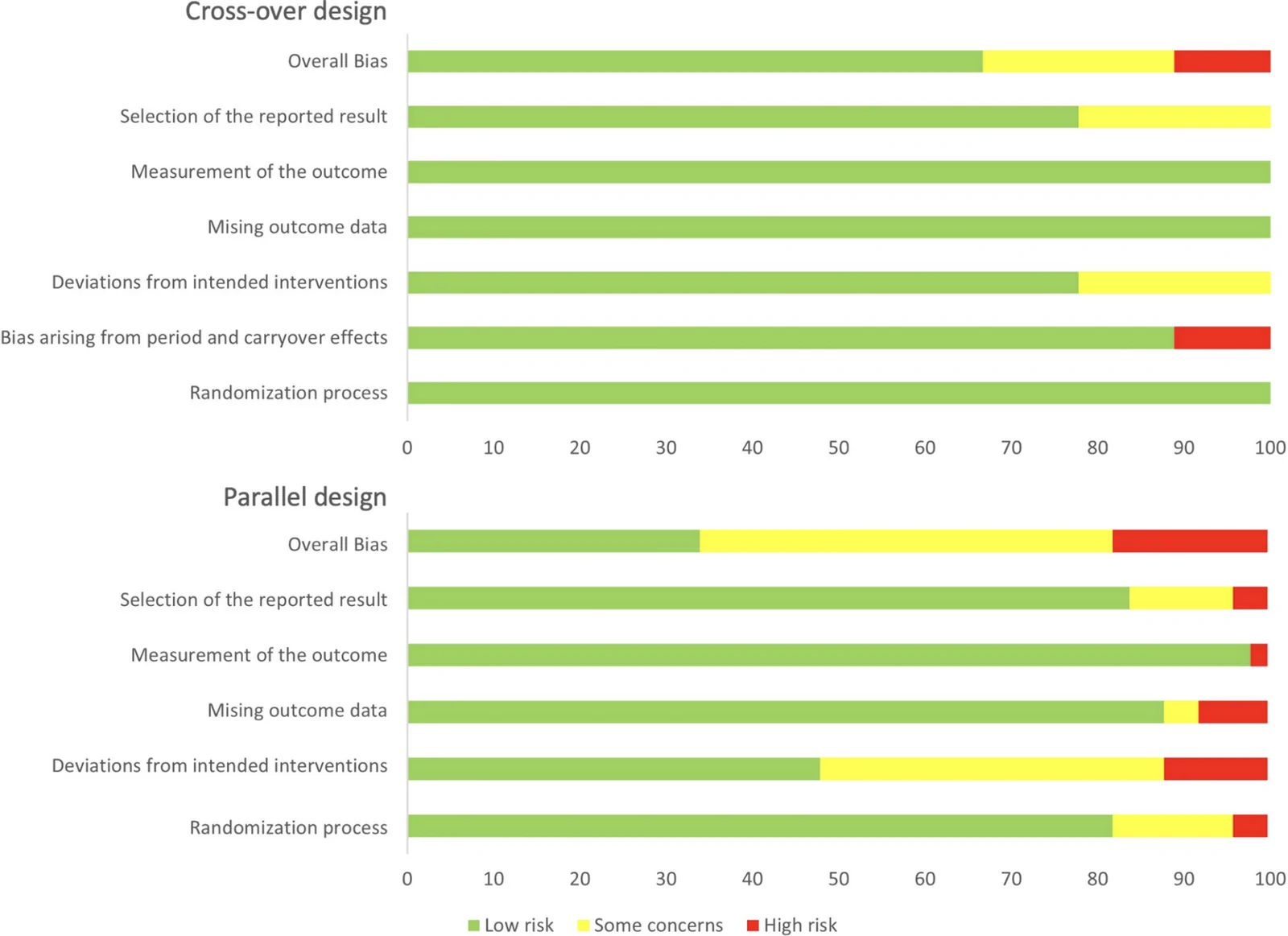
Summary of the assessment of risk of bias according to the revised Cochrane risk-of-bias tool for randomized trials (RoB 2)

According to the RoB 2 assessment, 23 studies were considered to have a low risk of bias, 26 had some concerns, and 10 trials had a high risk of bias (Fig. S2). However, when considering study design, more than half of the crossover trials had a low risk of bias, while only one third of parallel group trials had a similar assessment (Fig. 1). Besides, the majority of the studies (> 80%) with both study designs had low or some concerns, demonstrating an overall reliability of the evidence (Fig. 1).

### Effect of anthocyanin interventions on cognitive function

The meta-analysis of the effect of anthocyanin intervention on cognitive performance was based on 11 datasets for general cognition [30–40], 34 datasets for attention and visuospatial reasoning [30–35, 37, 40–56, 58–63, 63], 33 datasets for working memory [30–33, 37, 41–45, 47–49, 52, 56, 59–74], 30 datasets for episodic memory [30, 34, 35, 37, 41, 42, 47, 51–55, 59, 60, 63, 66, 69, 75–83], 41 datasets for processing and psychomotor speed [30, 31, 33–35, 37, 39, 41–43, 45–49, 51, 52, 54, 56–65, 67–69, 72, 77, 79, 84–88] and 15 datasets for verbal speed and fluency [30, 32–34, 37, 48, 51, 56, 58, 64, 65, 67, 76, 79, 80].

**Fig. 2:**
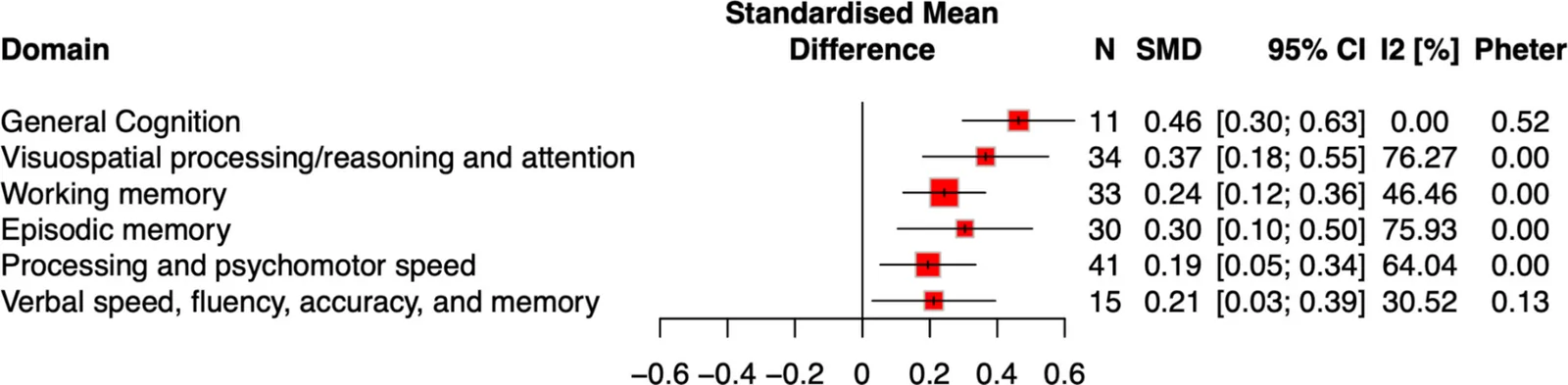
Summary results of the effect of anthocyanin intervention on general cognition and individual cognitive domains; *I*^2^ – statistic of heterogeneity (with *P*, *P* value for heterogeneity); SMD, standardized mean difference; CI, confidence interval; weight, weight assigned to each study based on random-effects model of meta-analysis

The summary results for all outcomes are presented in Fig. 2 with study-specific effect sizes provided in Fig. S3. All domains resulted significantly affected by the intervention: better cognitive functioning in favor of anthocyanins compared to the control was found for general cognition (SMD = 0.46, 95% CI = 0.30 to 0.63, *I*^2^ = 0.0%), visuospatial processing/reasoning and attention (SMD = 0.37, 95% CI = 0.18 to 0.55, *I*^2^ = 76.3%), processing and psychomotor speed (SMD = 0.19, 95% CI = 0.05 to 0.34, *I*^2^ = 64.0%) and for verbal speed and fluency (SMD = 0.21, 95% CI = 0.03 to 0.39, *I*^2^ = 30.5%), episodic memory (SMD = 0.30, 95% CI = 0.10 to 0.50, *I*^2^ = 75.9%), as well as for working memory (SMD = 0.24, 95% CI = 0.12 to 0.36, *I*^2^ = 46.5%, Fig. 2 and Fig. S3). After imputing different correlation coefficients (*r* = 0.2 and 0.8), the results did not change (data not shown). Based on the Egger test, evidence of publication bias was detected for visuospatial processing/reasoning and attention, working memory, episodic memory, and psychomotor speed (*P* < 0.05, Fig. S4). The trim-and-fill analysis adjusted for the number of missing studies showed similar results, except for visuospatial processing/reasoning and attention (SMD = 0.08, 95% CI = −0.13 to 0.29, *I*^2^ = 85.2%) and working memory (SMD = 0.08, 95% CI = −0.06 to 0.22, *I*^2^ = 66.5%) domains for which the effect of the intervention lost significant Table S4.

To check the robustness of the findings, sensitivity analyses were performed. Influential analysis in which one study at a time was excluded generally showed stable results for all cognitive domains (Fig. S5). The most substantial changes were noted after exclusion of (i) Carrillo et al., 2025 (SMD = 0.40, 95% CI = 0.22 to 0.58) and Calapai et al., 2017 (SMD = 0.41, 95% CI = 0.22 to 0.59) for general cognition, (ii) Cheatham et al., 2023 (SMD = 0.26, 95% CI = 0.13 to 0.39) for visuospatial processing/reasoning and attention, (iii) Calapai et al., 2017 (SMD = 0.18, 95% CI = −0.02 to 0.37), Amone et al., 2024 (SMD = 0.18, 95% CI = −0.01 to 0.37) and Flanagan et al., 2022 (SMD = 0.19, 95% CI = −0.01 to 0.37) for verbal speed and fluency, (iv) Kent et al., 2017 (SMD = 0.15, 95% CI = 0.03 to 0.28) and Bohn, 2021 (SMD = 0.15, 95% CI = 0.03 to 0.28) for processing and psychomotor speed (v) Bohn et al., 2021 (SMD = 0.23, 95% CI = 0.05 to 0.40), Krikorian et al., 2022 (SMD = 0.25, 95% CI = 0.06 to 0.43) and Chai et al., 2019 (SMD = 0.24, 95% CI = 0.06 to 0.40) for episodic memory and (vi) Calapai et al., 2017 (SMD = 0.07, 95% CI = −0.06 to 0.19) and Lee et al., 2017 (SMD = 0.15, 95% CI = 0.01 to 0.30) for working memory. Alternative analyses with selection of different psychological tests within each cognitive domain in each study also confirmed the stability of the findings **(**Fig. S6 and Table S5). In the three scenarios developed, most results remained significant, with an improvement in most cognitive domains after intervention with anthocyanins. In only 2 alternative analyses, the effects of the intervention did not result in significant improvements in episodic memory and verbal speed, fluency, accuracy, and memory (Fig. S6 and Table S5). Finally, a supplemental model meta-analyzing all tests for individual cognitive domains within each study and then pooling together the retrieved effect sizes showed substantially unchanged findings, although still with evidence of heterogeneity (Fig. S7). Additional sensitivity analyses were performed by excluding specific groups of studies according to a variety of criteria (Table S6).

All results generally remained unchanged after excluding studies in which a combined intervention was tested, as well as after restricting the analysis to studies with a berry product only (Table S6). However, when studies were restricted to those with an anthocyanin content of ≥ 10 mg/day, results remained significant for general cognition, visuospatial processing/reasoning and attention, verbal speed, fluency, accuracy, and memory. At the same time when studies were restricted to those with juice intervention, a significant association was found for visuospatial processing/reasoning and attention, working memory, episodic memory, and processing and psychomotor speed (Table S6).

Subgroup analysis of the effect of anthocyanin supplementation on cognitive performance generally did not reveal substantial differences by health status (p_subgr_ > 0.05) for general cognition; visuospatial processing/reasoning and attention; working memory; processing and psychomotor speed; verbal speed; fluency; accuracy; and memory; however, the estimates often lost significance in the subgroup of participants without mental or cognitive problems. On the contrary, in the episodic memory domain, the protective effect of intervention was only found in cognitively impaired populations. The intervention form did not affect the result for general cognition and the visuospatial processing/reasoning and attention domain; however, for the remaining domains, a tendency towards a stronger positive effect was detected in studies with anthocyanin supplementation by food compared to capsules, with a statistically significant difference in episodic memory as well as on the boundary of significance in the psychomotor speed domain (Table 2). Additionally, a trend towards a stronger beneficial effect was noted in longer trials (≥ 3 months *vs*. < 3 months) in general cognition, episodic memory, and especially in verbal speed, fluency, accuracy, and memory. At the same time, the results did not differ substantially by the mean age of participants (Table 2). The results of subgroup analysis by study design and daily dose of anthocyanin supplementation (≥ 100 mg *vs*. < 100 mg) showed no significant differences. However, due to the limited number of studies with crossover designs and available information on dose, the results should be interpreted with caution.

**Table 2:** Subgroup analysis for the effect of anthocyanin intervention on general cognition and individual cognitive domains

Grouping variable	Subgroup	*n*	SMD [95% CI]	*I*^2^	*P*_subgr_
*General cognition*					
Cognitive status of participants	With mental or cognitive problems^#^	5	0.52 [0.30; 0.74]***	20.9%	0.295
	Without mental or cognitive problems	5	0.30 [−0.04; 0.65]	0.0%	
Intervention form	Food	6	0.30 [0.03; 0.58]*	0.0%	0.178
	Capsules	5	0.55 [0.32; 0.78]***	13.8%	
Follow up	< 3 months	3	0.33 [−0.04; 0.70]	0.0%	0.493
	≥ 3 months	8	0.48 [0.28; 0.69]***	13.5%	
Mean age of participants	< 65y	6	0.44 [0.23; 0.65]***	0.0%	0.893
	≥ 65y	5	0.47 [0.15; 0.78]**	14.1%	
Design	Parallel	9	0.41 [0.22; 0.61]***	0.0%	0.571
	Crossover	2	0.57 [0.06; 1.08]*	63.2%	
Anthocyanin content	< 100 mg/d	4	0.31 [−0.03; 0.65]	0.0%	0.949
	≥ 100 mg/d	3	0.29 [−0.06; 0.65]	0.0%	
*Visuospatial processing/reasoning and attention*					
Cognitive status of participants	With mental or cognitive problems^#^	23	0.24 [0.10; 0.38]***	42.7%	0.139
	Without mental or cognitive problems	9	0.75 [0.09; 1.41]*	91.6%	
Intervention form	Food	23	0.42 [0.16; 0.69]**	79.4%	0.385
	Capsules	11	0.26 [0.02; 0.51]*	68.1%	
Follow up	< 3 months	9	0.33 [0.09; 0.56]**	27.4%	0.762
	≥ 3 months	25	0.38 [0.14; 0.61]**	81.2%	
Mean age of participants	< 65y	19	0.19 [0.03; 0.36]*	50.7%	0.036
	≥ 65y	14	0.67 [0.26; 1.07]**	86.2%	
Design	Parallel	30	0.39 [0.18; 0.60]***	79.0%	0.288
	Crossover	4	0.21 [−0.04; 0.47]	0.0%	
Anthocyanin content	< 100 mg/d	12	0.20 [0.07; 0.34]**	0.0%	0.492
	≥ 100 mg/d	12	0.37 [−0.08; 0.81]	88.1%	
*Working memory*					
Cognitive status of participants	With mental or cognitive problems^#^	24	0.24 [0.11; 0.38]***	47.4%	0.332
	Without mental or cognitive problems	6	0.10 [−0.16; 0.36]	33.0%	
Intervention form	Food	22	0.24 [0.11; 0.37]***	22.2%	0.966
	Capsules	11	0.23 [−0.01; 0.47]	69.2%	
Follow up	< 3 months	15	0.25 [0.07; 0.44]**	31.3%	0.914
	≥ 3 months	18	0.24 [0.07; 0.40]**	56.7%	
Mean age of participants	< 65y	16	0.21 [0.06; 0.37]**	40.4%	0.666
	≥ 65y	16	0.27 [0.06; 0.48]*	55.6%	
Design	Parallel	28	0.28 [0.14; 0.42]***	52.2%	0.127
	Crossover	5	0.07 [−0.15; 0.30]	0.0%	
Anthocyanin content	< 100 mg/d	8	0.05 [−0.09; 0.19]	0.0%	0.690
	≥ 100 mg/d	14	0.09 [−0.05; 0.23]	0.0%	
*Episodic memory*					
Cognitive status of participants	With mental or cognitive problems^#^	16	0.10 [−0.14; 0.33]	69.2%	0.020
	Without mental or cognitive problems	14	0.59 [0.24; 0.93]***	80.3%	
Intervention form	Food	18	0.57 [0.20; 0.94]**	82.2%	0.014
	Capsules	12	0.07 [−0.07; 0.21]	26.2%	
Follow up	< 3 months	5	0.30 [−0.50; 1.10]	89.6%	0.978
	≥ 3 months	25	0.29 [0.09; 0.49]**	70.6%	
Mean age of participants	< 65y	13	0.39 [0.06; 0.72]*	81.1%	0.484
	≥ 65y	17	0.24 [−0.02; 0.50]	71.7%	
Design	Parallel	28	0.34 [0.13; 0.55]**	76.7%	0.053
	Crossover	2	−0.18 [−0.68; 0.31]	29.7%	
Anthocyanin content	< 100 mg/d	10	0.00 [−0.16; 0.16]	0.0%	0.335
	≥ 100 mg/d	13	0.11 [−0.05; 0.28]	28.9%	
*Psychomotor speed*					
Cognitive status of participants	With mental or cognitive problems^#^	29	0.11 [0.01; 0.20]*	2.5%	0.189
	Without mental or cognitive problems	10	0.44 [−0.05; 0.93]	87.5%	
Intervention form	Food	27	0.29 [0.08; 0.50]**	70.4%	0.061
	Capsules	14	0.05 [−0.10; 0.19]	27.5%	
Follow up	< 3 months	19	0.22 [0.01; 0.43]*	59.6%	0.748
	≥ 3 months	22	0.17 [−0.02; 0.37]	68.0%	
Mean age of participants	< 65y	22	0.11 [−0.01; 0.23]	14.4%	0.187
	≥ 65y	18	0.33 [0.03; 0.63]*	80.3%	
Design	Parallel	32	0.22 [0.04; 0.39]*	69.8%	0.652
	Crossover	9	0.16 [−0.02; 0.34]	7.0%	
Anthocyanin content	< 100 mg/d	10	−0.03 [−0.19; 0.13]	0.0%	0.101
	≥ 100 mg/d	19	0.20 [−0.02; 0.41]	66.2%	
*Verbal speed, fluency, accuracy, and memory*					
Cognitive status of participants	With mental or cognitive problems^#^	8	0.27 [0.07; 0.48]**	27.4%	0.672
	Without mental or cognitive problems	6	0.20 [−0.09; 0.49]	0.0%	
Intervention form	Food	10	0.27 [0.06; 0.47]*	0.0%	0.399
	Capsules	5	0.07 [−0.34; 0.48]	70.9%	
Follow up	< 3 months	3	−0.18 [−0.73; 0.37]	63.5%	0.087
	≥ 3 months	12	0.32 [0.15; 0.49]***	0.0%	
Mean age of participants	< 65y	5	0.19 [−0.36; 0.73]	69.4%	0.953
	≥ 65y	10	0.21 [0.04; 0.38]*	0.0%	
Design	Parallel	14	0.21 [0.01; 0.41]*	35.3%	0.859
	Crossover	1	0.16 [−0.33; 0.66]	-	
Anthocyanin content	< 100 mg/d	5	0.21 [−0.11; 0.53]	29.0%	0.902
	≥ 100 mg/d	5	0.18 [−0.13; 0.49]	0.0%	

*P < 0.05; **P < 0.01; ***P < 0.001; *I*^2^ statistics of heterogeneity, *P*_heter_ *P* value for heterogeneity, *n* number of arms, *SMD* standardized mean difference, *P* *P* value for significance of the effect

## Discussion

The aim of this study was to summarize the current evidence on the mid- to long-term effects of anthocyanin supplementation on cognitive functioning in adults. The results of this meta-analysis showed that anthocyanin interventions significantly improved general cognition compared to the control. Importantly, similar results were observed for individual cognitive domains, including visuospatial processing/reasoning and attention, processing and psychomotor speed, verbal speed and fluency, episodic memory, and working memory. Most subgroup analyses did not reveal important confounding factors that may have weakened the robustness of results: some analyses did not result in significant findings possibly due to the lower number of studies included compared to the general meta-analysis, although the direction and strength of the effect remained unchanged. Notably, no relation between effect and anthocyanin dose was found, higher doses not being actually more effective than lower ones; in contrast, longer trials resulted in more effective improvements compared to shorter ones. These findings suggest that the intake of anthocyanin-rich foods may exert positive effects on health over a longer time consumption rather than excessive daily doses, which is in line with the biological rationale that food compounds are naturally designed to be consumed within certain dose boundaries and that may exert effects on health over an established chronic exposure. Overall, the results reported in this study support the hypothesis that plant-based dietary patterns rich in anthocyanins may be associated with better cognitive functioning and slower cognitive decline. Within this context, a higher share of anthocyanin-rich fruits, such as berries, may be important to justify such an outcome. Additionally, supplementing current diets with anthocyanin-rich products (i.e., juices or extracts) could be a potential strategy to improve cognitive status in older individuals.

Plant-based dietary patterns have recently gained increased attention as strategies to support healthy ageing, with particular relevance to mental and cognitive health. Among these, the Mediterranean diet has been extensively studied and consistently associated with a lower risk of cognitive decline, Alzheimer’s disease, and other forms of dementia [89]. This dietary pattern emphasizes the consumption of vegetables, fruits, legumes, whole grains, olive oil, and moderate intake of fish and wine [89]. However, while rich in general antioxidants and healthy fats, the traditional Mediterranean diet does not prominently feature anthocyanin-rich foods, such as berries, which are infrequently consumed in many Mediterranean populations [90]. To address this limitation and enhance the neuroprotective potential of dietary recommendations, the MIND diet (Mediterranean-DASH Intervention for Neurodegenerative Delay) has been proposed as a hybrid dietary model integrating core elements of the Mediterranean and DASH (Dietary Approaches to Stop Hypertension) diets while explicitly including anthocyanin-rich foods, particularly berries, as key components due to their emerging cognitive benefits: the inclusion of such foods in the MIND diet is grounded in growing evidence from epidemiological and interventional studies demonstrating that higher intake of berries and other flavonoid-rich foods is associated with slower cognitive ageing and reduced dementia risk [91]. In fact, longitudinal data show that greater adherence to the MIND diet is associated with a substantially lower incidence of Alzheimer’s disease, even among non-Mediterranean populations [92]. This suggests that adapting dietary models to include neuroprotective phytochemicals, such as anthocyanins, may provide added value beyond traditional patterns.

The mechanisms through which anthocyanins exert their cognitive benefits are multifactorial and involve several key processes. Anthocyanins have demonstrated potential benefits against vascular and endothelial damage in both observational [15] and intervention studies [93, 94]. Since vascular dysfunction and poor cerebral blood flow might be key contributors to cognitive decline, anthocyanins can preserve cognitive function by acting on endothelial function and promoting nitric oxide (NO) production, leading to enhanced blood flow to the brain [95]. By improving vascular health, anthocyanins support optimal oxygen and nutrient delivery to neurons, which is essential for maintaining cognitive function [96]. Anthocyanins can act as potent antioxidants, scavenging reactive oxygen species (ROS) and reducing oxidative damage to cellular components, including lipids, proteins, and DNA [97]. Oxidative stress has been implicated in the pathogenesis of Alzheimer's disease and other neurodegenerative disorders, and by neutralizing ROS, anthocyanins protect neurons from damage [98]. Notably, more complex mechanisms, such as modulation of microglia and astrocyte activity (the primary immune cells of the central nervous system) and inhibition of neuroinflammation have been suggested as more credible mechanisms of protection against cognitive decline and other neurodegenerative disorders [99]. Chronic inflammation is typically occurring in the ageing brain and in most neurodegenerative conditions [100]. Anthocyanins have been shown to modulate inflammatory pathways by reducing the activation of microglia and astrocytes through the inhibition of signaling pathways, such as NF-κB and MAPK, and reduction of pro-inflammatory cytokine production and neuronal damage [101]. Also, anthocyanins may enhance synaptic function and promote neurogenesis in the hippocampus, a brain region critical for memory and spatial navigation [102]. Synaptic plasticity has been shown to be crucial for learning and memory formation. This effect may be mediated by the upregulation of brain-derived neurotrophic factor (BDNF), a key neurotrophic factor involved in the survival and growth of neurons [103]. Notably, such mechanisms are supported by the recognition of biomarkers also in human studies, resulting in a pro-inflammatory cytokine profile and signaling molecules of vascular damage [104, 105].

Current experimental models elucidating the mechanisms behind the gut-brain axis are evolving towards more holistic and complex methodologies, incorporating omics approaches to describe the relationship between diet and brain health [106]. The main concerns regarding the evidence of the potential benefits of anthocyanins on human health stem from the entire process of absorption and metabolism, as only a minor fraction of the compounds is actually absorbed and remains unchanged within the human body [107]. The mechanisms governing the absorption, metabolism, and excretion of (poly)phenols are complex and involve several stages, including processes that occur in the gastrointestinal tract, liver, kidneys, and various organs, with significant contributions from gut microbiota [108]. While the observed antioxidant properties have been demonstrated for the native compounds, current evidence suggests that anthocyanins undergo extensive transformation by the gut microbiota, leading to the production of a variety of metabolites that may exert other effects in the human brain [17]. In fact, anthocyanins reaching the colon are transformed by the gut microbiota into their aglycone forms (the non-glycosylated, sugar-free forms), which are more readily available for further degradation by other microbes into other compounds, including phenolic acids, which have demonstrated neuroprotective and pro-cognitive activities through different molecular mechanisms including the modulation of pro-oxidant and antioxidant machinery as well as inflammatory status [109]. On the other hand, anthocyanin intake has been shown in both in vitro and in vivo studies to beneficially modulate the gut microbiota. These compounds increase the abundance of beneficial bacterial groups, such as *Bifidobacterium*, *Lactobacillus*, *Faecalibacterium prausnitzii*, and *Eubacterium rectale*, while reducing potentially harmful bacteria like *Clostridium spp.*, *Desulfovibrio*, *Enterococcus*, and others [110]. These shifts are associated with increased production of short-chain fatty acids (SCFAs), particularly butyrate, which supports gut barrier integrity, reduces inflammation, and contributes to metabolic and systemic health [111, 112]. Anthocyanins also counteract dysbiosis and enhance microbial diversity through their prebiotic-like actions, promoting a richer and more balanced microbiome [113]. The changes in gut microbiota induced by anthocyanin intake could benefit brain health through the gut-brain axis by enhancing populations of SCFA-producing bacteria (e.g., *Bifidobacterium*, *Faecalibacterium*) which support neuroprotection, reduce systemic and neuroinflammation, and promote BBB integrity [114]. Moreover, certain anthocyanin metabolites and SCFAs may act as signaling molecules, influencing neurotransmitter synthesis, modulating microglial activity, and supporting cognitive function [115]. Therefore, the microbial shifts promoted by anthocyanins may partly explain their observed effects on mood regulation, cognitive performance, and reduced risk of neurodegenerative disorders. Hence, it is unclear whether the observed effects rely unequivocally on the direct action of anthocyanins in the brain, or rather on an indirect action of the gut microbiota.

The results of the present meta-analysis should be considered in light of some limitations. Firstly, the relative heterogeneity of the included studies regarding the age and health status of participants, as well as the characteristics of the intervention (including foods/drinks, or extracts), and trial duration, could weaken the results. However, several sensitivity analyses were conducted to overcome this limitation. Secondly, the potential interactions, including accumulating, synergistic, and antagonistic effects, with other unknown dietary components as well as food matrices, cannot be ruled out. Also, the difference in the actual exposure to anthocyanin metabolites resulting from gut microbiota composition was not accounted for. Finally, several aspects investigated in the original studies using different tests and grouped in the present study within a certain group may, in part, overlap in clinical significance and physiological involvement of similar brain structures: for instance, attention tests may also include a reaction time and a motor response, hence considered in the present study as testing for “processing and psychomotor time”, but in fact they might be considered within the group testing for attention; also memory speed could be deemed as attention, although it has its basis on episodic memory, and so many other situations in which the categorization of the outcomes might suffer from arbitrariness. However, this meta-analysis is the first one providing alternative models, including various combinations of tests, to consider potential less favorable alternative scenarios, in order to reduce possible bias and explore the stability of the results.

In conclusion, the results indicate that anthocyanin intake improves cognitive functioning in adults. The results of the present meta-analysis suggest that future RCTs should adopt moderate daily content of anthocyanin but last for at least 4 months or more. Future studies could further strengthen current evidence possibly accounting for interindividual differences, identifying markers of consumption, gut microbiota modifications related to cognitive function, and metabolites exerting direct effects in the brain. Emerging tools, such as metabolomics or microbiome profiling, could be integrated into future RCTs to identify responders or anthocyanin-derived bioactive metabolites and gut bacterial population profiling in order to distinguish metabotypes and other unmeasured variables that might explain interindividual variability among participants.

## Supplementary Information

Below is the link to the electronic supplementary material.

ESM 1 (DOCX 6.33 MB)

## Data Availability

Not applicable.
