# Correction to: The effect of anthocyanins and anthocyanin‑rich foods on cognitive function: a meta‑analysis of randomized controlled trials

**DOI:** 10.1007/s11357-026-02105-1

**Published:** 2026-03-24

**Authors:** Agnieszka Micek, Justyna Godos, Francesca Giampieri, Maurizio Battino, José L. Quiles, Daniele Del Rio, Pedro Mena, Giuseppe Caruso, Evelyn Frias‑Toral, Irma Domínguez Azpíroz, Jianbo Xiao, Nicola Veronese, Mario Siervo, David Vauzour, Zoltan Ungvari, Fabio Galvano, Giuseppe Grosso

**Affiliations:** 1 Statistical Laboratory, Faculty of Health Sciences, Jagiellonian University Medical College, Cracow, Poland; 2 Department of Biomedical and Biotechnological Sciences, University of Catania, Catania, Italy; 3 Department of Clinical Sciences, Universita Politecnica Delle Marche, Ancona, Italy; 4 Joint Laboratory On Food Science, Nutrition and Intelligent Processing of Foods, Polytechnic University of Marche, Ancona, Italy; 5 Universidad Europea del Atlantico, Spain and Jiangsu University, China at Polytechnic University of Marche, Ancona, Italy; 6 Research Group On Food, Nutritional Biochemistry and Health, Universidad Europea del Atlantico, Santander, Spain; 7 International Joint Research Laboratory of Intelligent Agriculture and Agri‑Products Processing, Jiangsu University, Zhenjiang, China; 8 Department of Physiology, Institute of Nutrition and Food Technology “Jose Mataix Verdu”, Biomedical Research Centre, University of Granada, Armilla, Spain; 9 Human Nutrition Unit, Department of Food and Drug, University of Parma, Parma, Italy; 10 Departmental Faculty of Medicine, UniCamillus-Saint Camillus International University of Health and Medical Sciences, Rome, Italy; 11 IRCCS San Camillo Hospital, Venice, Italy; 12 School of Medicine, Universidad Espiritu Santo, Samborondon, Ecuador; 13 Division of Research, Texas State University, San Marcos, TX USA; 14 Universidade Internacional Do Cuanza, Cuito, Angola; 15 Universidad de La Romana, La Romana, Dominican Republic; 16 Department of Analytical Chemistry and Food Science, Faculty of Food Science and Technology, University of Vigo, Ourense, Spain; 17 Curtin‑Chulalongkorn Collaborative Centre for Nutrition and Food Research and Education, Curtin University, Perth, WA Australia; 18 Faculty of Health Sciences, School of Population Health, Curtin University, Perth, WA Australia; 19 Curtin Dementia Centre of Excellence, Enable Institute, Curtin University, Perth, WA Australia; 20 Curtin Medical Research Institute (CMRI), Curtin University, Perth, WA Australia; 21 Norwich Medical School, Faculty of Medicine and Health Sciences, University of East Anglia, Norwich, UK; 22 Vascular Cognitive Impairment, Neurodegeneration and Healthy Brain Aging Program, Department of Neurosurgery, University of Oklahoma Health Sciences Center, Oklahoma City, OK USA; 23 Stephenson Cancer Center, University of Oklahoma, Oklahoma City, OK USA; 24 Oklahoma Center for Geroscience and Healthy Brain Aging, University of Oklahoma Health Sciences Center, Oklahoma City, OK USA; 25 Department of Health Promotion Sciences, College of Public Health, University of Oklahoma Health Sciences Center, Oklahoma City, OK USA; 26 International Training Program in Geroscience, Doctoral College/Institute of Preventive Medicine and Public Health, Semmelweis University, Budapest, Hungary

**Correction to: GeroScience**

10.1007/s11357-025-02008-7

In this article, the consortium details in the author group were incorrectly given as “International Network for Evidence on Phytochemicals, Biotics for Human Health” but should have been “International Network for Evidence on Phytochemicals and Biotics for Human Health”.

The original article has been corrected.

The publisher sincerely apologizes for this error and any inconvenience caused to the authors and readers of the journal.

