# Supplementary material for: The effect of anthocyanins and anthocyanin-rich foods on cognitive function: a meta-analysis of randomized controlled trials
Source: GeroScience. 2025 Dec 6;48(5):6929–63. doi: 10.1007/s11357-025-02008-7 (PMC13601464; doi:10.1007/s11357-025-02008-7)
Supplement: Supplementary file 1 [file 11357_2025_2008_MOESM1_ESM.docx]

The effect of anthocyanins and anthocyanin-rich foods on cognitive function: a meta-analysis of randomized controlled trials

Geroscience

Agnieszka Micek^1^, Justyna Godos^2^, Francesca Giampieri^3,4,5,6^, Maurizio Battino^3,4,5,7^, José L Quiles^8^, Daniele Del Rio^9^, Pedro Mena^9^, Giuseppe Caruso^10,11^, Evelyn Frias-Toral^12,13^, Irma Domínguez Azpíroz^4,14,15^, Jianbo Xiao^16^, Nicola Veronese^17^, Mario Siervo^18,19,20,21^, David Vauzour^22^, Zoltan Ungvari^23,24,25,26,27^, Fabio Galvano^2^, Giuseppe Grosso^2^

1 Statistical Laboratory, Faculty of Health Sciences, Jagiellonian University Medical College, 31-501, Cracow, Poland;

2 Department of Biomedical and Biotechnological Sciences, University of Catania, Catania, Italy;

3 Department of Clinical Sciences, Università Politecnica delle Marche, 60131 Ancona, Italy;

4 Research Group on Food, Nutritional Biochemistry and Health, Universidad Europea del Atlántico, Isabel Torres 21, 39011 Santander, Spain;

5 Joint Laboratory on Food Science, Nutrition, and Intelligent Processing of Foods, Polytechnic University of Marche, Italy, Universidad Europea del Atlántico Spain and Jiangsu University, China at Polytechnic University of Marche, 60130 Ancona, Italy;

6 International Research Center for Food Nutrition and Safety, Jiangsu University, Zhenjiang 212013, China;

7 International Joint Research Laboratory of Intelligent Agriculture and Agri-Products Processing, Jiangsu University, Zhenjiang 212013, China;

8 Department of Physiology, Institute of Nutrition and Food Technology ''José Mataix Verdú", Biomedical Research Centre, University of Granada, Avda. del Conocimiento s.n., 18100 Armilla, Spain;

9 Human Nutrition Unit, Department of Food and Drug, University of Parma, Parma, 43125, Italy;

10 Departmental Faculty of Medicine, UniCamillus-Saint Camillus International University of Health and Medical Sciences, Rome, Italy;

11 IRCCS San Camillo Hospital, Venice, Italy;

12 School of Medicine, Universidad Espíritu Santo, Samborondón 0901952, Ecuador;

13 Division of Research, Texas State University, 601 University Dr, San Marcos, TX 78666, USA;

14 Universidade Internacional do Cuanza, Cuito EN250, Angola;

15 Universidad de La Romana, La Romana 22000, Dominican Republic;

16 Department of Analytical Chemistry and Food Science, Faculty of Food Science and Technology, University of Vigo, Ourense Campus, E-32004 Ourense, Spain;

17 Saint Camillus International University of Health Sciences, Rome, Italy;

18 Curtin-Chulalongkorn Collaborative Centre for Nutrition and Food Research and Education, Curtin University, Perth, WA, Australia and Chulalongkorn University, Bangkok, Thailand;

19 Faculty of Health Sciences, School of Population Health, Curtin University, Perth, WA 6102, Australia;

20 Curtin Dementia Centre of Excellence, Enable Institute, Curtin University, Perth, WA 6102, Australia;

21 Curtin Medical Research Institute (CMRI), Curtin University, Perth, WA 6102, Australia;

22 Norwich Medical School, Faculty of Medicine and Health Sciences, University of East Anglia, Norwich NR4 7TJ, United Kingdom;

23 Vascular Cognitive Impairment, Neurodegeneration and Healthy Brain Aging Program, Department of Neurosurgery, University of Oklahoma Health Sciences Center, Oklahoma City, OK, USA;

24 Stephenson Cancer Center, University of Oklahoma, Oklahoma City, OK, USA;

25 Oklahoma Center for Geroscience and Healthy Brain Aging, University of Oklahoma Health Sciences Center, Oklahoma City, OK, USA;

26 Department of Health Promotion Sciences, College of Public Health, University of Oklahoma Health Sciences Center, Oklahoma City, OK, USA;

27 International Training Program in Geroscience, Doctoral College/Institute of Preventive Medicine and Public Health, Semmelweis University, Budapest, Hungary.

Corresponding author: Giuseppe Caruso, giuseppe.caruso@unicamillus.org

Table S1. Preferred Reporting Items for Systematic Reviews and Meta-Analyses (PRISMA) checklist

| **Section/topic** | **#** | **Checklist item** | **Reported on page #** |
| --- | --- | --- | --- |
| **TITLE** | | |  |
| Title | 1 | Identify the report as a systematic review, meta-analysis, or both. | 1 |
| **ABSTRACT** | | |  |
| Structured summary | 2 | Provide a structured summary including, as applicable: background; objectives; data sources; study eligibility criteria, participants, and interventions; study appraisal and synthesis methods; results; limitations; conclusions and implications of key findings; systematic review registration number. | 3 |
| **INTRODUCTION** | | |  |
| Rationale | 3 | Describe the rationale for the review in the context of what is already known. | 4,5 |
| Objectives | 4 | Provide an explicit statement of questions being addressed with reference to participants, interventions, comparisons, outcomes, and study design (PICOS). | 5 |
| **METHODS** | | |  |
| Protocol and registration | 5 | Indicate if a review protocol exists, if and where it can be accessed (e.g., Web address), and, if available, provide registration information including registration number. | 5 |
| Eligibility criteria | 6 | Specify study characteristics (e.g., PICOS, length of follow-up) and report characteristics (e.g., years considered, language, publication status) used as criteria for eligibility, giving rationale. | 6,7 |
| Information sources | 7 | Describe all information sources (e.g., databases with dates of coverage, contact with study authors to identify additional studies) in the search and date last searched. | 5,6 |
| Search | 8 | Present full electronic search strategy for at least one database, including any limits used, such that it could be repeated. | Table S2 |
| Study selection | 9 | State the process for selecting studies (i.e., screening, eligibility, included in systematic review, and, if applicable, included in the meta-analysis). | Figure S1 |
| Data collection process | 10 | Describe method of data extraction from reports (e.g., piloted forms, independently, in duplicate) and any processes for obtaining and confirming data from investigators. | 7 |
| Data items | 11 | List and define all variables for which data were sought (e.g., PICOS, funding sources) and any assumptions and simplifications made. | 7 |
| Risk of bias in individual studies | 12 | Describe methods used for assessing risk of bias of individual studies (including specification of whether this was done at the study or outcome level), and how this information is to be used in any data synthesis. | 7 |
| Summary measures | 13 | State the principal summary measures (e.g., risk ratio, difference in means). | 7,8 |
| Synthesis of results | 14 | Describe the methods of handling data and combining results of studies, if done, including measures of consistency (e.g., I^2^) for each meta-analysis. | 7,8 |
| Risk of bias across studies | 15 | Specify any assessment of risk of bias that may affect the cumulative evidence (e.g., publication bias, selective reporting within studies). | 7,8 |
| Additional analyses | 16 | Describe methods of additional analyses (e.g., sensitivity or subgroup analyses, meta-regression), if done, indicating which were pre-specified. | 7,8 |
| **RESULTS** | | |  |
| Study selection | 17 | Give numbers of studies screened, assessed for eligibility, and included in the review, with reasons for exclusions at each stage, ideally with a flow diagram. | 8 |
| Study characteristics | 18 | For each study, present characteristics for which data were extracted (e.g., study size, PICOS, follow-up period) and provide the citations. | Table 1 |
| Risk of bias within studies | 19 | Present data on risk of bias of each study and, if available, any outcome level assessment (see item 12). | Figure S2, Figure 1 |
| Results of individual studies | 20 | For all outcomes considered (benefits or harms), present, for each study: (a) simple summary data for each intervention group (b) effect estimates and confidence intervals, ideally with a forest plot. | Figure S3 |
| Synthesis of results | 21 | Present results of each meta-analysis done, including confidence intervals and measures of consistency. | Figure 2 |
| Risk of bias across studies | 22 | Present results of any assessment of risk of bias across studies (see Item 15). | Figure S4 |
| Additional analysis | 23 | Give results of additional analyses, if done (e.g., sensitivity or subgroup analyses, meta-regression [see Item 16]). | Figure S5, Figure S6, Table 2, Table S4-S6 |
| **DISCUSSION** | | |  |
| Summary of evidence | 24 | Summarize the main findings including the strength of evidence for each main outcome; consider their relevance to key groups (e.g., healthcare providers, users, and policy makers). | 11-14 |
| Limitations | 25 | Discuss limitations at study and outcome level (e.g., risk of bias), and at review-level (e.g., incomplete retrieval of identified research, reporting bias). | 14,15 |
| Conclusions | 26 | Provide a general interpretation of the results in the context of other evidence, and implications for future research. | 15 |
| **FUNDING** | | |  |
| Funding | 27 | Describe sources of funding for the systematic review and other support (e.g., supply of data); role of funders for the systematic review. | 15 |

Table S2. Systematic search strategy

| PubMed search | |
| --- | --- |
| 1 | anthocyanin*[Title/Abstract] OR bilberry[Title/Abstract] OR strawberry[Title/Abstract] OR blueberr*[Title/Abstract] OR blackcurrant[Title/Abstract] OR "Aronia melanocarpa"[Title/Abstract] OR "Concord grape"[Title/Abstract] OR pomegranate[Title/Abstract] OR cherr*[Title/Abstract] OR berr*[Title/Abstract] OR elderberr*[Title/Abstract] OR plum*[Title/Abstract] OR grape*[Title/Abstract] |
| 2 | cognitive[Title/Abstract] OR cognition[Title/Abstract] OR memory[Title/Abstract] |
| 3 | intervention*[Title/Abstract] OR supplement*[Title/Abstract] OR trial*[Title/Abstract] OR random*[Title/Abstract] OR placebo[Title/Abstract] OR control*[Title/Abstract] OR RCT[Title/Abstract] OR parallel[Title/Abstract] OR "cross over"[Title/Abstract] OR "clinical trial*"[publication type] OR "randomized controlled trial"[publication type] OR "Clinical Trials as Topic"[MeSH] |
| 4 | 1 AND 2 AND 3 |

Table S3. Population, Intervention, Comparison, Outcomes and Study (PICOS) criteria

| **Parameter** | **Description** |
| --- | --- |
| (P) Population | Adults men and non-pregnant women, excluding patients with end-stage degenerative diseases |
| (I) Intervention | Anthocyanin or anthocyanin-rich food supplementation |
| (C) Comparison | Placebo or other comparator that did not contain examined polyphenol or that contains it in a small amount |
| (O) Outcome | Psychological tests assessing: general cognition, attention and visuospatial reasoning, working memory, episodic memory, processing and psychomotor speed, verbal speed and fluency |
| (S) Study design | Randomised controlled trials |

Table S4. Effect of anthocyanin intervention on general cognition and individual cognitive domains: results of trim-and-fill analysis

| **Domain** |  | **n** | **SMD [95% CI]** | **I^2^** | **P_heter_** |
| --- | --- | --- | --- | --- | --- |
| General cognition |  | 14 | 0.51 [0.35; 0.68]*** | 5.8% | 0.389 |
| Visuospatial processing/ reasoning and attention |  | 45 | 0.08 [-0.13; 0.29] | 85.2% | <0.001 |
| Working memory |  | 43 | 0.08 [-0.06; 0.22] | 66.5% | <0.001 |
| Episodic memory |  | - | - | - | - |
| Psychomotor speed |  | - | - | - | - |
| Verbal speed, fluency, accuracy, and memory |  | 17 | 0.26 [0.06; 0.46]* | 43.9% | 0.027 |
| *P<0.05, **P<0.01, ***P<0.001; I^2^ – statistics of heterogeneity, P_heter_ – p value for heterogeneity, n – number of arms, SMD – standardised mean difference, P – p value for significance of the effect | | | | | |

Table S5. Summary results for the effect of anthocyanin intervention on general cognition and individual cognitive domains in alternative models based on selection of different psychological tests

| **Domain** | **n** | **Alternative 1** | **Alternative 2** | **Alternative 3** |
| --- | --- | --- | --- | --- |
|  |  | **SMD [95% CI]** | **SMD [95% CI]** | **SMD [95% CI]** |
| General Cognition | 11 | 0.29 [0.03; 0.54]* | 0.48 [0.31; 0.65]*** | 0.38 [0.16; 0.59]*** |
| Visuospatial processing/ reasoning and attention | 34 | 0.27 [0.08; 0.45]** | 0.43 [0.24; 0.62]*** | 0.27 [0.09; 0.44]** |
| Working memory | 33 | 0.17 [0.04; 0.29]* | 0.38 [0.24; 0.52]*** | 0.22 [0.10; 0.34]*** |
| Episodic memory | 30 | 0.29 [-0.02; 0.60] | 0.34 [0.15; 0.54]*** | 0.22 [0.05; 0.39]** |
| Psychomotor speed | 41 | 0.14 [0.03; 0.25]* | 0.31 [0.19; 0.43]*** | 0.14 [0.03; 0.26]* |
| Verbal speed, fluency, accuracy, and memory | 15 | 0.24 [0.03; 0.45]* | 0.53 [0.24; 0.82]*** | 0.31 [-0.03; 0.65] |
| *P<0.05, **P<0.01, ***P<0.001; n – number of arms, SMD – standardised mean difference, P – p value for significance of the effect | | | | |

Table S6. Sensitive analysis of the effect of anthocyanin intervention on general cognition and individual cognitive domains after exclusion of selected studies

| **Cognitive domain** |  | **n** | **SMD [95% CI]** | **I^2^** |
| --- | --- | --- | --- | --- |
| **General cognition** | Exclusion of studies that tested a combined intervention | 10 | 0.46 [0.29; 0.63]*** | 0.70% |
|  | Restriction to studies with berry product intervention only | 9 | 0.41 [0.22; 0.60]*** | 0% |
|  | Restriction to studies with juice intervention only | 4 | 0.28 [-0.04; 0.59] | 0% |
|  | Restriction to studies with information on anthocyanin content available ≥10 mg/day | 7 | 0.30 [0.05; 0.55]* | 0% |
| **Visuospatial processing/ reasoning and attention** | Exclusion of studies that tested a combined intervention | 34 | 0.37 [0.18; 0.55]*** | 76.30% |
|  | Restriction to studies with berry product intervention only | 27 | 0.39 [0.16; 0.61]*** | 80.60% |
|  | Restriction to studies with juice intervention only | 12 | 0.67 [0.18; 1.15]** | 87.90% |
|  | Restriction to studies with information on anthocyanin content available ≥10 mg/day | 24 | 0.29 [0.07; 0.51]** | 77.60% |
| **Working memory** | Exclusion of studies that tested a combined intervention | 33 | 0.24 [0.12; 0.36]*** | 46.50% |
|  | Restriction to studies with berry product intervention only | 27 | 0.27 [0.13; 0.41]*** | 50.70% |
|  | Restriction to studies with juice intervention only | 11 | 0.30 [0.09; 0.51]** | 39.40% |
|  | Restriction to studies with information on anthocyanin content available ≥10 mg/day | 21 | 0.09 [-0.01; 0.20] | 0.00% |
| **Episodic memory** | Exclusion of studies that tested a combined intervention | 29 | 0.29 [0.09; 0.49]** | 76.20% |
|  | Restriction to studies with berry product intervention only | 26 | 0.27 [0.08; 0.47]** | 72.30% |
|  | Restriction to studies with juice intervention only | 7 | 1.04 [0.30; 1.78]** | 84.30% |
|  | Restriction to studies with information on anthocyanin content available ≥10 mg/day | 20 | 0.07 [-0.05; 0.18] | 1.20% |
| **Processing and psychomotor speed** | Exclusion of studies that tested a combined intervention | 39 | 0.19 [0.04; 0.34]* | 65.70% |
|  | Restriction to studies with berry product intervention only | 32 | 0.15 [0.00; 0.29]* | 57.00% |
|  | Restriction to studies with juice intervention only | 15 | 0.53 [0.20; 0.86]** | 77.30% |
|  | Restriction to studies with information on anthocyanin content available ≥10 mg/day | 29 | 0.12 [-0.03; 0.27] | 55.50% |
| **Verbal speed, fluency, accuracy, and memory** | Exclusion of studies that tested a combined intervention | 14 | 0.21 [0.01; 0.40]* | 35.50% |
|  | Restriction to studies with berry product intervention only | 11 | 0.22 [-0.01; 0.44] | 37.40% |
|  | Restriction to studies with juice intervention only | 4 | 0.15 [-0.15; 0.45] | 0.00% |
|  | Restriction to studies with information on anthocyanin content available ≥10mg/day | 10 | 0.20 [0.00; 0.40]* | 0.00% |
| *P<0.05, **P<0.01, ***P<0.001; I^2^ – statistics of heterogeneity, n – number of arms, SMD – standardised mean difference, P – p value for significance of the effect | | | | |

Figure S1. Flow chart of the study selection process

Figure S2. Assessment of risk of bias according to the revised Cochrane risk-of-bias tool for randomized trials (RoB 2)

Figure S3 Forest plots for the effect of anthocyanin intervention on A) General cognition, B) Working memory, C) Processing and psychomotor speed, D) Attention and visuospatial reasoning, E) Episodic memory, F) Verbal speed and fluency; I2 – statistic of heterogeneity (with p – p value for heterogeneity), SMD – standardised mean difference, CI – confidence interval, Weight – weight assigned to each study based on random-effects model of meta-analysis

Figure S4. Funnel plots for the effect of anthocyanin intervention on general cognition and individual cognitive domains. Abbreviations: GenCogn - General cognition; Attention - Attention and visuospatial reasoning; WorkMem -Working memory; EpisMem - Episodic memory; PsychSpeed - Processing and psychomotor speed; VerbFluen - Verbal speed and fluency. Black dots represent studies included in meta-analysis, empty dots refer to missing studies added in trim-and-fill analysis. Presented p-values are expressed in format p1//p2, where p1 refers to p from Egger’s regression test before and p2 after trim-and-fill analysis

Figure S5 Forest plots for the effect of anthocyanin intervention on general cognition and individual cognitive domains: A) General cognition, B) Working memory, C) Processing and psychomotor speed, D) Attention and visuospatial reasoning, E) Episodic memory, and F) Verbal speed and fluency; I2 – statistic of heterogeneity (with p – p value for heterogeneity), SMD – standardised mean difference, CI – confidence interval, Weight – weight assigned to each study based on random-effects model of meta-analysis

Figure S6 Forest plots of the three alternative analyses for the effect of anthocyanin intervention on general cognition and individual cognitive domains: A) General cognition, B) Working memory, C) Processing and psychomotor speed, D) Attention and visuospatial reasoning, E) Episodic memory, and F) Verbal speed and fluency; I2 – statistic of heterogeneity (with p – p value for heterogeneity), SMD – standardised mean difference, CI – confidence interval, Weight – weight assigned to each study based on random-effects model of meta-analysis

Alternative analysis 1

Figure S6 Forest plots of the three alternative analyses for the effect of anthocyanin intervention on general cognition and individual cognitive domains: A) General cognition, B) Working memory, C) Processing and psychomotor speed, D) Attention and visuospatial reasoning, E) Episodic memory, and F) Verbal speed and fluency; I2 – statistic of heterogeneity (with p – p value for heterogeneity), SMD – standardised mean difference, CI – confidence interval, Weight – weight assigned to each study based on random-effects model of meta-analysis

Alternative analysis 2

Figure S6 Forest plots of the three alternative analyses for the effect of anthocyanin intervention on general cognition and individual cognitive domains: A) General cognition, B) Working memory, C) Processing and psychomotor speed, D) Attention and visuospatial reasoning, E) Episodic memory, and F) Verbal speed and fluency; I2 – statistic of heterogeneity (with p – p value for heterogeneity), SMD – standardised mean difference, CI – confidence interval, Weight – weight assigned to each study based on random-effects model of meta-analysis

Alternative analysis 3

Figure S7 Figure S7 Sensitivity analysis for the effect of anthocyanin intervention on general cognition and individual cognitive domains by averaging the results of all tools used for individual domains in each study and meta-analyzing the summary effect sizes
